# Evidence of air pollution-related ocular signs and altered inflammatory cytokine profile of the ocular surface in Beijing

**DOI:** 10.1038/s41598-022-23294-7

**Published:** 2022-11-01

**Authors:** Dalan Jing, Xiaodan Jiang, Peng Zhou, Xiaotong Ren, Jie Su, Ran Hao, Mingzhong Zhang, Yu Wan, Xuemin Li

**Affiliations:** 1 Department of Ophthalmology, Peking University Third Hospital, Beijing, People’s Republic of China; 2 Beijing Key Laboratory of Restoration of Damaged Ocular Nerve, Peking University Third Hospital, 49 North Garden Rd., Haidian District, Beijing, 100191 People’s Republic of China

**Keywords:** Biochemistry, Environmental sciences, Environmental social sciences, Diseases, Signs and symptoms

## Abstract

We evaluated how different degrees of air pollution affect the ocular surface of a cohort of human subjects in Beijing by correlating in-patient test outcomes with tear cytokines. A cross-sectional study involving 221 volunteers was carried out in different districts of Beijing. Air pollution indices were recorded for 7 d (including the visit day). The indices recorded were the air quality index (AQI), which is a dimensionless measure that quantitatively describes the state of air quality, concentrations of particulate matter smaller than 2.5 μm (PM2.5) and 10 μm (PM10), sulfur dioxide (SO_2_), ozone (O_3_), and nitrogen dioxide (NO_2_). The Ocular Symptom Disease Index (OSDI) questionnaire provided. Subsequently, subjects underwent slit-lamp examination, which included meibomian gland examination, conjunctival congestion score, conjunctivochalasis grade, tear meniscus height (TMH), tear breakup time (TBUT), corneal fluorescein staining (CFS), Schirmer I test, and conjunctival impression cytology. The concentrations of vascular endothelial growth factor (VEGF), interleukins (IL)-1β, IL-6 and IL-8 in tears were measured by microsphere-based immunoassay analysis. According to the value of the AQI, participants are divided into a slightly polluted (SP) group (n = 103) which the AQI value is less than or equal to 100 and a heavily polluted (HP) group (n = 118) whose AQI value is more than 100. Air pollution is related to ocular discomfort based on tear cytokine concentrations. PM2.5, PM10 and NO_2_ were positively correlated with OSDI, MG expressibility, meibum score, meiboscore, conjunctival congestion score, Schirmer I test value, TMH, goblet-cell density, concentrations of IL-6, and VEGF were negatively correlated with TBUT. PM2.5 and PM10 appear to be the major risk factors to the ocular surface, with NO_2_ being another important risk factor based on this study. The symptoms and signs of eye discomfort in the SP group were significantly less severe than those in the HP group, and tear cytokine concentrations (IL-6 and VEGF) were lower. Air pollution degrees were significantly correlated with tear cytokine concentrations, indicating an alteration of cytokine balance at the ocular surface under different degrees of air pollution.

## Introduction

In recent decades, rapid industrialisation and urbanisation have brought not only economic development but also unprecedented air pollution^1,2^. Ambient air pollution is a complex of particulate matter (PM) (solid and liquid particles suspended in air) and gases e.g., ozone, NO_2_ and SO_2_, that constitute the dimensions of World Health Organisation air quality assessment^2–4^. Exposure to air pollution poses an urgent challenge to public health because it is everywhere, affects everyone, and has numerous severe adverse effects on the respiratory and cardiovascular systems^5–8^. Similar to the respiratory system, the ocular surface is constantly exposed to the external environment, which is also affected by air pollution^9^.

Air pollution is associated with several eye diseases, including conjunctivitis, dry eye disease and age-related macular degeneration^10^. Residents of heavily polluted areas experience more severe ocular discomfort^11,12^. Decreased tear film stability, influenced tear film osmolarity and goblet cell hyperplasia have been detected on ocular surfaces of individuals living in areas with high degrees of environmental air pollution^9,13–15^. Numerous studies both in vivo and in vitro have demonstrated the upregulation of cytokines on the ocular surface after exposure to air pollution. Tau^16^ found that human corneal and conjunctival epithelial cells incubated with diesel exhaust particles showed cytotoxicity and an inflammatory response mediated by the cytokine IL-6. Li^17^ considered rat animal model exposure to airborne carbon black that induced corneal fluorescein staining (CFS) increased and upregulation of IL-4 and interferon (IFN)-γ in the anterior segment. Activation of inflammatory pathways and oxidative stress are considered potential mechanisms involved in the pathogenesis of ocular surface diseases after air pollution exposure^18,19^.

Despite these observations, the mechanism of the adverse effects of air pollution on the ocular surface is still unclear. This study aimed to measure the concentrations of various tear cytokines in individuals exposed to different degrees of air pollution (utilizing AQI) and to analyse correlations between these degrees and clinical test outcomes during ocular surface in-patient examination. Because the ocular surface discomfort caused by air pollution is similar to dry eye, we selected four inflammatory cytokines related to dry eye. Although air pollution affects the whole eyeball, our focus is on the ocular surface because it is easy to observe and evaluate.

## Materials and methods

### Subjects

We studied 221 healthy volunteers (221 eyes) who were recruited in different regions of Beijing with different pollution degrees from January 2019 to December 2019. The number of volunteers recruited in each region was as follows (in order of increasing air quality index [AQI]: Miyun District, 39 people; Yanqing District, 64 people; Haidian District, 44 people; Chaoyang District, 51 people; Tongzhou District, 18 people; Daxing District, 5 people). The time for recruiting volunteers is concentrated in the heating period in Beijing. Miyun and Yanqing districts located on the periphery of Beijing had lower population density, fewer motor vehicles, and higher forest density compared to Haidian and Chaoyang districts which located in the close suburb. Tongzhou and Daxing districts were operating large industrial waste gas and automobile exhaust emissions^20–23^. The inclusion criteria were as follows: human subjects who had lived in the corresponding district for at least one year and a place of residence and work within a 5 km radius from the environmental atmospheric monitoring station of this study. All subjects were systemically healthy, not pregnant, noncontact lens users, not under any medication, and had no previous history of ophthalmic disease (e.g., allergic conjunctivitis, retinal disease, cataracts, etc.). Open men and women in each group, aged between 18 and 80 year. We routinely enrolled the right eye. If the right eye did not meet the inclusion criteria, the left eye was enrolled instead.

The study was approved by the institutional review board/ethics committee of the Peking University Third Hospital (M2019101) and is in accordance with the Declaration of Helsinki. Informed consent was obtained from all participants.

### Collection of air quality data

Air component pollution data (PM2.5, PM10, SO_2_, NO_2_, and O_3_) of regions were obtained from the China Environmental Monitoring Station. Previous monitoring methods and instruments were used to measure air pollutant data^24,25^. Daily average concentrations of PM2.5 and PM10 were determined by a tapered element oscillating microbalance (TEOM). SO_2_ and NO_2_ were determined by ultraviolet fluorescence and chemiluminescence. O_3_ was determined by a nondispersive ultraviolet fluorescence photometer^25,26^. The AQI, which is designed to integrate various pollutant concentrations into a single daily value and to reflect the comprehensive status of air quality, was also calculated. The AQI may not be a perfect guide that correlates with ocular surface health. Grades 1 and 2 should not be definitive of minimal adverse health effects but can crudely be thought of as having good air quality. When the grade greater than 2, symptoms of susceptible people are slightly aggravated, and irritation symptoms appear in healthy people (Supplementary document 3). The AQI calculation method and classification criteria in China follow the AQI definition^27^. The process of AQI calculation and evaluation can be roughly divided into three steps: the first step is to calculate the individual air quality index (IAQI) based on the measured concentration values of PM2.5, PM10, SO_2_, NO_2_, O_3_, CO and other pollutants by comparing the hierarchical concentration limits of various pollutants; the second step is to select the maximum value from the IAQI of various pollutants and determine it as the AQI. When the AQI is greater than 50, the pollutant with the largest IAQI is determined as the primary pollutant. The third step is to determine the air quality level, category, color, health impact and recommended measures according to the AQI classification standard. (Detailed classification is shown in Supplementary material.) In order to truly study the impact of air pollution on the eye surface and avoid the impact of drastic changes in AQI on the test, air pollution data collection was performed for an average of 7 d, including the inspection day. By the definition of the AQI^28^, grades 1 and 2 represent good air quality with minimal adverse effects on individuals, we included individuals at grades 1 and 2 in the SP group (n = 103). However, when the AQI is greater than grade 2, the normal population and susceptible people have irritation symptoms or aggravation of symptoms. Thus, we included individuals with grades greater than 2 in the HP group (n = 118).

### Clinical examination

All individuals were evaluated using the OSDI questionnaire, which was translated into Chinese^29^. The clinical assessments of the enrolled subjects were conducted in the following order: collection of demographic information (including age and sex) and eye signs, including conjunctival injection^30^, conjunctivochalasis grade^31^, tear meniscus height (TMH)^32^, tear breakup time (TBUT)^33^, CFS^34^, Schirmer I test value^35^, and meibomian gland (MG) assessments^36^ (including MG expressibility and meibum score). Subsequently, impression cytology^37^ obtained a sample in the superficial layers of the inferior tarsal conjunctiva by adhesion to cellulose-acetate filters; subsequently, the adhered tissue was stained for histological analysis. A keratograph (Oculus Optikgeräte GmbH, Wetzlar, Germany) was used to observe MG morphology, and the meiboscore was defined as described by Arita^38^. An interval of 5 min was required between different examinations. Ophthalmologic examinations were performed and assessed by the same ophthalmologist.

### Tear collection

Tear collection was performed before any other test and was limited to a maximum of 10 min. All tear samples were collected between 10 and 11 AM. Nonirritating tear collection was conducted without anesthesia by using 5-µL capillary pipettes. At least 50 µL of tears were collected. The sample was then transferred into 0.2-mL Eppendorf tubes (Axygen, USA) and stored at −80 °C until further analysis.

### Measurement of inflammatory cytokines

Tear samples were analysed for cytokine concentrations (pg/mL) using a published protocol^39,40^. This method involves multiplex cytometric bead array (multiplex-CBA) for quantitative analysis of IL-1β, IL-6, IL-8, and VEGF (Human ICAM Plex Flex Set, Human IL-6 Plex Flex Set, Human IL-8 Plex Flex Set, Human VEGF Plex Flex Set kits, respectively; BD Biosciences, San Diego, CA, USA). Multiplex-CBA was performed according to the manufacturer's instructions. A tear volume of 50 μL per eye was used for each analysis. All samples were processed in the same analytical run. Data were acquired on a BD FACSCanto II flow cytometer (Becton Dickinson, Franklin Lakes, NJ, USA) and analysed using BD FCAP Array software (Becton Dickinson, Franklin Lakes, NJ, USA).

### Data analysis

All analyses were performed using SPSS software (version 25.0). We verified the normality of the data distribution using the Kolmogorov‒Smirnov test. Descriptive parameters are expressed as the number of patients (%) or mean ± standard deviation/median with interquartile range, depending on the distribution pattern. Pearson and Spearman tests were used for correlation analysis. Statistical significance for intergroup differences was assessed by the nonparametric Wilcoxon signed-rank test and Mann–Whitney *U* test. Statistical significance was set at *P* value < 0.05. To avoid the correlation between these eye parameters, the real correlation between air quality parameters and eye parameters will be affected. Partial correlation analysis was performed to assess the independence of effective factors. Then, a stepwise regression procedure was performed for multiple correlations.

### Ethics approval and consent to participate (human ethics)

Approved from the institute’s ethics committee. Written informed consent was obtained from all subjects.

## Results

### Subject demographics

A total of 221 subjects with a mean and median age of 58.79 and 63 years, respectively, were recruited in six districts. Of these, 103 cases were included in the SP group, and 118 were included in the HP group. There were no significant differences among the groups regarding age (SP: median age 63 [46, 70]; HP: median age 64 [58, 71];* p* = 0.32) or sex (SP: 66% female, 34% male; HP: 86% female, 32% male; *p* = 0.27). The correlations between the degrees of air component pollution and AQI were evaluated, and a higher concentration of PM2.5 was positively correlated with the AQI (r = 0.92, p < 0.01) compared with the other air components. (See supplementary document 2 for details).

### Ocular characteristics of the subjects

**Figure 1:**
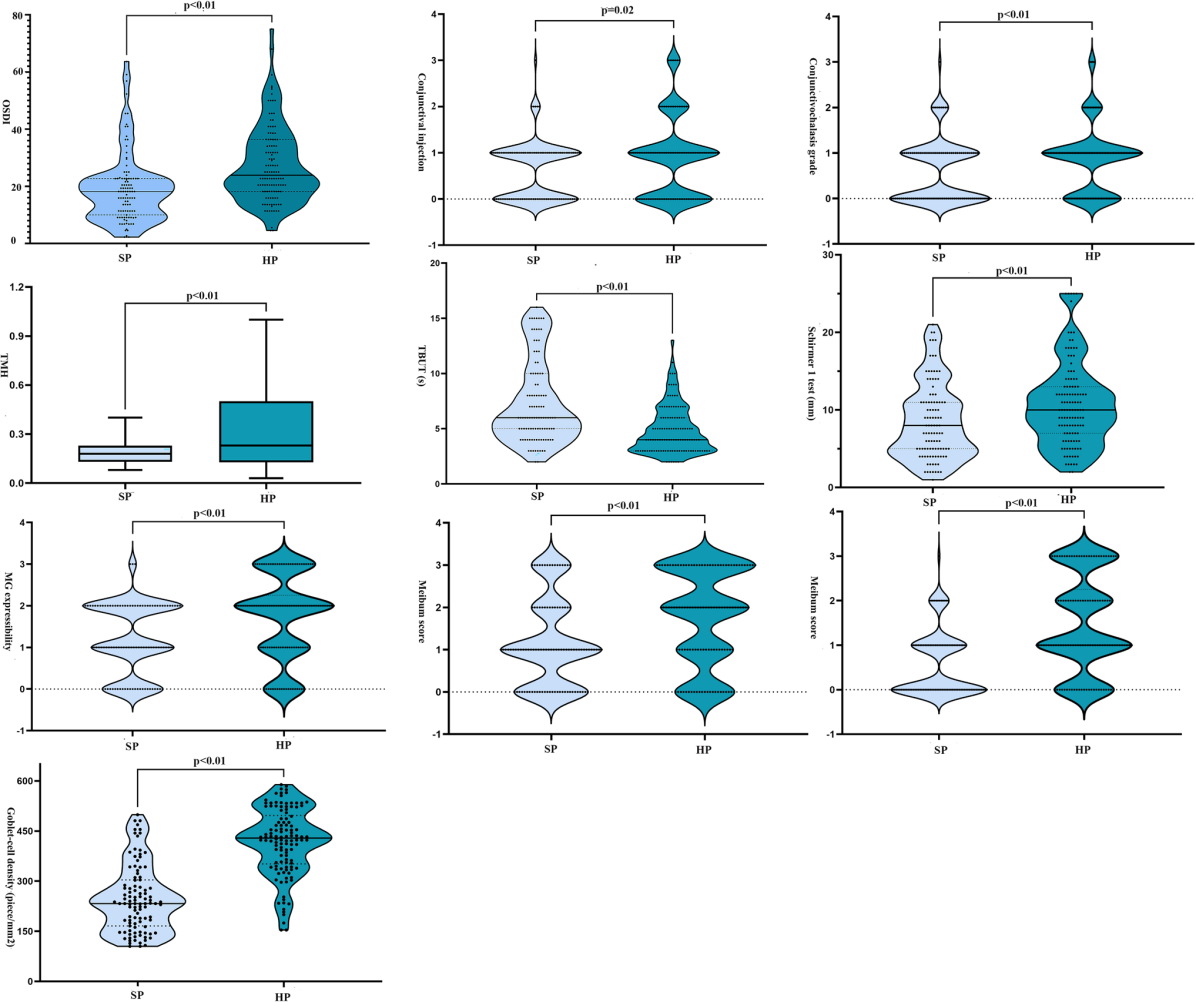
Comparison of ocular characteristics between the slightly (SP) and heavily polluted (HP) groups.

The ocular characteristics of the SP and HP subjects are summarised in Fig. 1. A significant difference in all eye examinations (*p* < 0.05), including OSDI scores, MG expressibility, meibum score, meiboscore, conjunctival injection, conjunctivochalasis grade, Schirmer I test, TBUT, TMH and goblet cell density, but no significant differences in CFS score (*p* = 0.97) were observed between these groups.

### Tear cytokine concentrations

**Figure 2:**
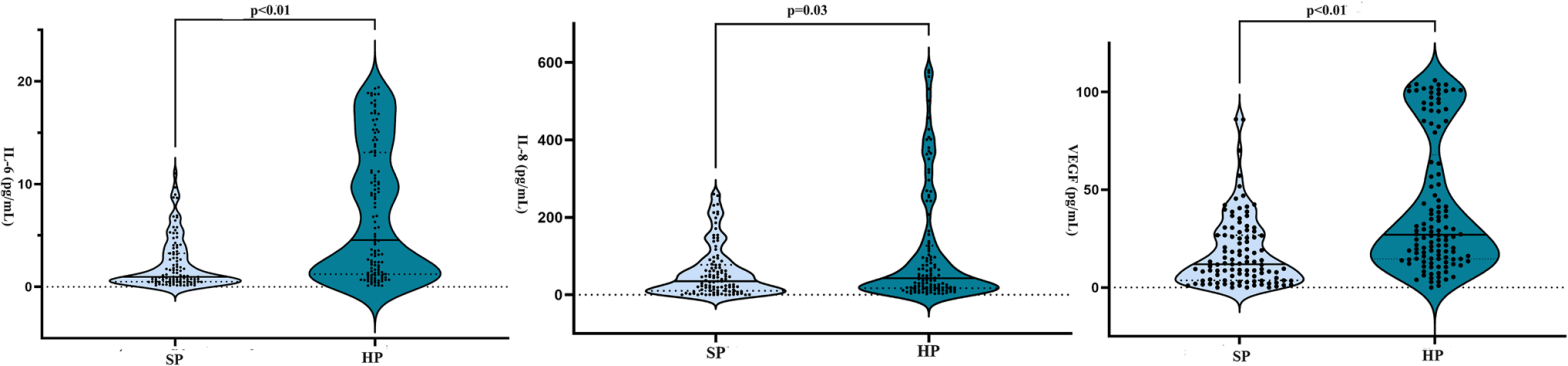
Cytokine concentrations in tears from the SP and HP groups determined by multiplex cytometric bead array.

The concentrations of IL-1β, IL-6, IL-8, and VEGF in the tear films of each group are shown in Fig. 2***. ***The HP group had significantly increased concentrations of IL-6 (*p* < 0.01), IL-8 (*p* = 0.03), and VEGF (*p* < 0.01) compared to the SP group.

### Correlations between air pollution and tear cytokines

The correlations of cytokine concentrations with air pollution, OSDI questionnaire scores and ocular signs are summarised in Table 1. The concentration of IL-1β was not significantly correlated with the AQI, PM2.5, PM10 or NO_2_ concentration. The IL-6 concentration was positively correlated with the AQI, PM2.5, PM10 and NO_2_ concentrations and negatively correlated with the concentrations of O_3_. IL-8 was positively correlated with AQI, PM2.5, PM10 and NO_2_ concentrations. VEGF concentration was positively correlated with AQI, PM2.5, PM10, SO_2_ and NO_2_ concentrations. However, it was negatively correlated with O_3_ concentration. However, partial correlation analysis revealed that there was no significant correlation between the concentrations of O_3_ and IL-6. The IL-8 concentration had no correlation with the AQI, PM2.5, PM10, or NO_2_ concentration.

**Table 1:** Correlations between air pollution data and all ocular parameters.

Ocular parameters	AQI		PM2.5		PM10		SO_2_		NO_2_		O_3_	
	r	Corrected r	r	Corrected r	r	Corrected r	r	Corrected r	r	Corrected r	r	Corrected r
OSDI	0.439**	0.229**	0.451**	0.201**	0.456**	0.232**	0.081	–	0.312**	0.165**	− 0.178**	–
MG expressibility	0.245**	0.245**	0.207**	0.207**	0.220**	0.220**	0.184**	0.184**	0.151*	0.151*	0.052	–
Meibum score	0.210**	0.210**	0.141*	0.141*	0.135*	0.135*	0.134*	0.134*	0.134*	0.134*	0.022	–
Meiboscore	0.387**	0.387**	0.350**	0.350**	0.361**	0.361**	0.146*	0.146*	0.290**	0.290**	− 0.067	–
conjunctival injection	0.296**	0.296**	0.266**	0.266**	0.264**	0.264**	0.131	–	0.251**	0.251**	− 0.087	–
Conjunctivochalasis grade	0.191**	0.191**	0.108	–	0.134*	0.134*	0.076	–	0.059	–	0.160*	0.160*
CFS score	0.009	–	− 0.054	–	− 0.071	–	0.015	–	− 0.099	–	0.252**	0.252**
Schirmer I test	0.189**	0.189**	0.222**	0.222**	0.214**	0.214**	0.124	–	0.142*	0.142*	− 0.097	–
TBUT	− 0.398**	− 0.257**	− 0.367**	− 0.192**	− 0.306**	− 0.165**	− 0.143*	− 0.141*	− 0.239**	− 0.132**	0.189**	–
TMH	0.254**	0.392**	0.280**	0.386**	0.265**	0.372**	0.146*	0.159*	0.301**	0.346**	− 0.189**	− 0.135**
Goblet-cell density	0.683**	0.370**	0.586**	0.221**	0.595**	0.247**	0.285**	− 0.173**	0.444**	0.226**	− 0.236**	–
IL-1β	− 0.046	–	− 0.017	–	− 0.041	–	− 0.021	–	0.019	–	− 0.061	–
IL-6	0.465**	0.309**	0.448**	0.252**	0.451**	0.338**	− 0.035	–	0.288**	0.164**	− 0.210**	–
IL-8	0.259**	–	0.247**	–	0.219**	–	0.032	–	0.156*	–	− 0.040	–
VEGF	0.556**	0.378**	0.539**	0.360**	0.538**	0.369**	0.159*	0.191*	0.462**	0.346**	− 0.305**	− 0.182**

OSDI, ocular symptom disease index; MG, Meibomian gland; CFS, corneal fluorescein staining; TBUT, tear breakup time; TMH, tear meniscus height; IL-1β, Interleukin-1β; IL-6, Interleukin-6; IL-8, Interleukin-8; VEGF, vascular endothelial growth factor; AQI, air quality index; PM2.5, particulate matter smaller than 2.5 μm; PM10, particulate matter smaller than 10 μm; SO_2_, oxidation of sulfur dioxide; NO_2_, nitrogen dioxide; O_3_, ozone.

*Significant correlation (*P* < 0.05).

**Significant correlation (*P* < 0.01).

–, no correlation was detected during correlation analysis.

### Correlations among air pollution, ocular symptoms and signs

Table 1 summarises the relationship between the concentrations of PM2.5, PM10, SO_2_, NO_2_, O_3_ and AQI and all eye parameters, including the OSDI score, conjunctival injection, TMH, TBUT, CFS score, Schirmer I test value, MG assessments and goblet cell density. The OSDI score showed a positive correlation with AQI, PM2.5, PM10 and NO_2_ concentrations but a negative correlation with O_3_ concentration. MG expressibility, Meibum score and Meiboscore were positively correlated with AQI, PM2.5, PM10, SO_2_ and NO_2_ concentration. Conjunctival injection was positively correlated with AQI, PM2.5, PM10 and NO_2_ concentrations. Conjunctivochalasis grade was associated with AQI, PM10 and O_3_ concentration. The CFS score was positively correlated with the O_3_ concentration. The Schirmer I test was positively correlated with AQI, PM2.5, PM10 and NO_2_ concentrations. In addition, TBUT was negatively correlated with AQI, PM2.5, PM10, SO_2_ and NO_2_ concentrations but positively correlated with O_3_ concentrations. TMH was positively correlated with the AQI, PM2.5, PM10, SO_2_ and NO_2_ concentrations and negatively correlated with the O_3_ concentration. Goblet-cell density was positively correlated with AQI, PM2.5, PM10 and NO_2_ concentrations and negatively correlated with O_3_ concentrations. Partial correlation analysis revealed that the O_3_ concentration was not correlated with the OSDI score, TBUT, or goblet cell density. For other results, these results have been shown to be consistent with normal correlation analysis, but correlation coefficients decreased. Furthermore, we used linear regression analysis to describe the relationship between PM2.5 concentration and OSDI scores. The linear equation best fitting the data were y = 0.05 x + 19.12 (R2 = 0.12, *p* < 0.01), where y is the OSDI score and x is the PM2.5 concentration.

### Correlations between tear cytokines and eye parameters

**Figure 3:**
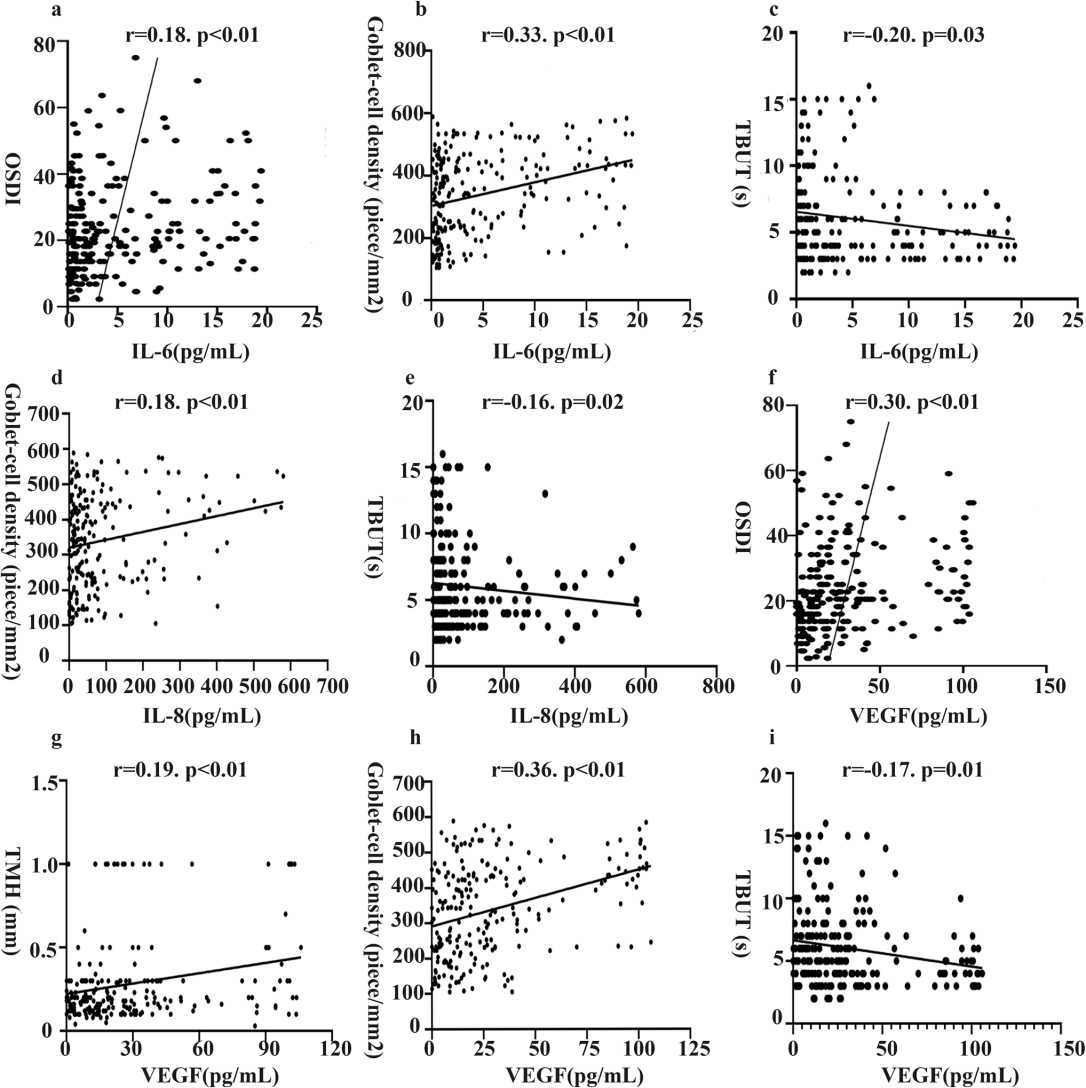
Correlations between cytokine concentrations and ocular surface parameters.

The correlations between the concentrations of IL-1β, IL-6, IL-8 and VEGF and ocular surface parameters, including OSDI score, conjunctival injection, conjunctivochalasis grade, TMH, TBUT, CFS, Schirmer I test value, MG assessments and goblet cell density, were evaluated. The concentration of IL-1β was negatively correlated with conjunctivochalasis grade (r = -0.17, *p* = 0.01). The concentration of IL-6 was positively correlated with the OSDI score (r = 0.18, *p* < 0.01; Fig. 3a) and goblet cell density (r = 0.33, *p* < 0.01; Fig. 3b) and negatively correlated with TBUT (r = -0.20, *p* = 0.03; Fig. 3c). The IL-8 concentration was positively correlated with conjunctival injection (r = 0.19, *p* < 0.01) and goblet cell density (r = 0.18, *p* < 0.01; Fig. 3d) and negatively correlated with TBUT (r = -0.16, *p* = 0.02; Fig. 3e). The VEGF concentration was positively correlated with the OSDI score (r = 0.30, *p* < 0.01; Fig. 3f), conjunctival injection (r = 0.23, *p* < 0.01), TMH (r = 0.19, *p* < 0.01; Fig. 3g), meiboscore (r = 0.24, *p* < 0.01) and goblet cell density (r = 0.36, *p* < 0.01; Fig. 3h) and was negatively correlated with TBUT (r = -0.17, *p* = 0.01; Fig. 3i). IL-1β, IL-6, IL-8, VEGF and other ocular surface parameters showed no statistical significance.

## Discussion

Previous studies reported that exposure of the ocular surface to high degrees of air pollution causes ocular discomfort, including burning, redness, and lacrimation^11,41^. Our findings of increased OSDI scores in the HP group agree with those of previous studies^42^. Except for ocular discomfort, abnormal ocular signs were also increased in high AQI. In our study, lower TBUT, TMH and Schirmer I test values were found in the HP group, consistent with the results of previous investigations^9,43^. This study found that subjects in highly polluted regions have increased MG expressibility, meibum score and meiboscore, which substantiates the findings of previous epidemiologic studies^44–46^. Some studies have indicated that fine particulate air pollution might influence MG function not only by absorbing the lipid of tear film but also by entering MG, leading to lipid deficiency or maldistribution^47,48^. These abnormalities may explain the instability of tear film. In addition, we found an increased goblet-cell density in subjects residing in heavily polluted areas, which is consistent with the finding in a study by Priscila^14^. Conjunctival goblet cells are slow-cycling cells that can proliferate in response to chronic inflammatory stimuli^49^, which implies that the mechanisms of adverse effects of air pollution on the ocular surface cause inflammatory and immune responses. Tau1^16^ demonstrated that conjunctival human epithelial cells incubated with diesel exhaust particles (DEPs) had increased expression of mucin genes (*MUC1, MUC5AC, and MUC16*). A significant increase in goblet cells and Mucosubstancia might produce an adaptive process that contributes to the protection of conjunctiva from adverse effects of air pollution.

Partial correlation analysis revealed a significant correlation between the AQI and eye parameters. Similarly, PM2.5 and PM10 were significantly correlated with eye parameters. Combined with those of regression analysis, these findings suggested that PM2.5 and PM10 are the main pollutants in air of what has been measured and have greater harm to the eye. According to previous studies on PM in respiratory system, PM10 particles deposit mainly in the upper respiratory tract, while PM2.5, known as fine particulate matter, can enter the lung alveoli easily and is retained in the body. Due to its small particle size, strong activity, ease of attachment to toxic and harmful substances, long persistence in the atmosphere and long transport distance. PM2.5 has a relatively large impact on human health and atmospheric environmental quality^50^. The PM2.5 concentration had a more significant positive correlation with the AQI than pollution. This suggested that the PM2.5 concentration may be partially representative of air quality when concentrations of other pollutants are unavailable.

A significant difference in the concentrations of inflammatory cytokines between the two groups was observed, also demonstrating that increasing air pollution degrees can cause ocular inflammatory reactions, which leads to significantly elevated tear cytokines. Using cultured conjunctival epithelial cells obtained from healthy people, Fujishima^51^ found that exposure to DEP increased the expression of IL-6 and intercellular adhesion molecules. In addition, an in vitro study suggested that inflammation caused by the DEP response is mediated by IL-6 and not by tumor necrosis factor α (TNF-α) or IL-8^16^. Lee^52^ reported increased concentrations of IL-1β, IL-6, IL-17, and interferon gamma in a murine model exposed to ozone chambers for one or two weeks. Matsuda^53^ showed that a high PM2.5 concentration is associated with a decrease in IL-5 and IL-10 concentrations. Taken together, these studies suggest that the inflammatory response plays an important role in the response of the ocular surface to air pollution. Similar mechanisms might be involved in air pollution-induced ocular changes as well as in dry eye disease (DED). Air pollution has also been implicated as a cause of DED^9,54^. Previous studies reported significantly higher concentrations of the tear inflammatory mediators IL-1β, IL-6, IL-8, and VEGF in the tears of patients with DED^55–57^. Increased tear cytokines may be the cause of irritation symptoms and ocular surface disease in DED^58^. Symptoms of DED are similar to ocular discomfort due to high air pollution^59^. Thus, we chose DED-related examinations and cytokines in our study. Inconsistent with the results of a previous report^44^, there was no difference in CFS between the groups in this study. This may be explained by our recruitment of healthy subjects with normal corneal integrity and fast recovery.

In correlation analysis, the OSDI was found to be correlated with IL-6 and VEGF concentrations, suggesting that these cytokines may be the cause of ocular discomfort. Concentrations of IL-6, IL-8, and VEGF were significantly associated with TBUT and goblet cell density, which indicates that these cytokines play an important role in ocular discomfort related to air pollution. A significantly positive correlation was observed between VEGF concentration and meiboscore. We speculate that exposure to air pollution results in ocular inflammatory responses, followed by increased cytokine concentrations and the absence of MG. To repair MG, the ocular surface induces VEGF production and enhances repair of the vascular system.

As air pollution increased, ocular discomfort occurred, and the OSDI score increased. Air pollution degrees (especially the PM2.5) were positively associated with MG score, conjunctival injection, TMH, and Schirmer I test value and negatively associated with TBUT. A significantly positive correlation was observed between PM2.5 and the concentrations of VEGF and IL-6. One possible explanation is that individuals living in heavily polluted areas may present an increased inflammatory response of the ocular surface. The concentrations of IL-6 and VEGF in tears increase with increasing PM2.5 concentration. The conjunctiva, irritated by inflammation and air pollutants, can be congested. Then, ocular discomfort occurs, and tears due to irritation are secreted to wash out pollutants and dilute inflammatory cytokines. Hence, the TMH and Schirmer I test values were elevated. As previously mentioned earlier, MG expressibility and secretion are altered, which may lead to a reduced lipid layer of tear film, further leading to instability of tear film. Long-term stimulation of inflammation and pollutants might result in goblet cell hyperplasia^14^. Then, mucin production is enhanced to protect the ocular surface. VEGF has a well-known angiogenic role^60^. In addition, VEGF expression in tears is increased in some ocular chronic inflammatory diseases, such as vernal keratoconjunctivitis (VKC) and DED, which are probably involved in the pathogenesis of conjunctival inflammation, remodelling and corneal changes in VKC^56,57^. VEGF can act as a direct proinflammatory mediator during the pathogenesis of rheumatoid arthritis^63^. Then, we hypothesised that VEGF may play a similar role in air pollution-related ocular discomfort. IL-6 contributes importantly to the pathophysiology of DED. The IL-6 concentration correlated significantly with various tear film and ocular surface parameters^64^. Tears from dry eye patients contain significantly increased concentrations of cytokines that show correlation to severity of the disease. Similarly, the upregulation of IL-6 and VEGF suggest that these cytokines play an important role in air pollution-related ocular discomfort. Topical cytokine modulators may be explored as a therapeutic approach to treat related discomfort. In fact, our study found correlations between IL-8 concentration and ocular parameters and suggested that IL-8 is not a major component of the ocular inflammatory pathway in air pollution, which is consistent with a recent report by Tau^65^.

This study is the first many-sided study of the effects of air pollution on ocular surface-related inflammation, symptoms and signs. The protective measures for people against air pollution may therefore include not only masks but also goggles.

Despite these findings, there are limitations in this study. First, we recruited only elderly individuals to reduce the effects of age on ocular inflammation, symptoms and signs. Future research may include the recruitment of participants of different ages. Second, while it is best to be able to use a personal contaminant monitor, it is unrealistic and costly. We only separately evaluated the impact of various pollutants on the ocular surface, but in real life, PM is a mixture of tens of thousands of chemical species, and they work together to cause changes in the ocular surface. And the sources of aerosols in different districts. We did not discuss these aspects. Third, the correlation coefficients among PM2.5, IL-6 and VEGF concentrations were small but of importance because the mechanism of ocular surface damage caused by air pollution is similar to but not completely the same as that of dry eye. Future research should include more types of inflammatory cytokines to explore the mechanism. Based on the results of the effects of pollutants on the ocular surface parameters, whether it is possible to redefine a new AQI to evaluate the effects of air pollutants on the ocular surface, which is different from the effects on the heart or lung. Moreover, gene expressions are more concrete evidence of mechanisms compared to protein concentrations. Future research should focus more on gene expression.

In this study, we found increased IL-6 and VEGF concentrations in subjects living in heavily polluted areas. The air pollution degrees, especially PM2.5, were significantly correlated with tear cytokine concentrations. Therefore, tear cytokines may be an effective approach to characterise the human response to ambient degrees of air pollution and alead to a novel breakthrough in treatment.

## Supplementary Information

Supplementary Information.

## Data Availability

All data generated or analyzed during this study are included in this published article.
