# Supplementary material for: Evidence of air pollution-related ocular signs and altered inflammatory cytokine profile of the ocular surface in Beijing
Source: Sci Rep. 2022 Nov 1;12:18359. doi: 10.1038/s41598-022-23294-7 (PMC9626484; doi:10.1038/s41598-022-23294-7)
Supplement: Supplementary file 1 — Supplementary Information. [file 41598_2022_23294_MOESM1_ESM.docx]

| District | n | AQI | PM2.5(µg/m^3^) | PM10(µg/m^3^) | O_3_ (µg/m^3^) | SO_2_ (µg/m^3^) | NO_2_ (µg/m^3^) |
| --- | --- | --- | --- | --- | --- | --- | --- |
| Miyun | 39 | 33.29±11.31 | 20.97±14.12 | 26.12±7.64 | 62.88±32.59 | 18.62±23.78 | 16.74±13.95 |
| Yanqing | 64 | 63.55±9.59 | 33.13±15.27 | 56.98±14.36 | 93.50±20.23 | 12.30±10.74 | 28.25±13.40 |
| Haidian | 44 | 104.29±4.24 | 62.86±6.06 | 108.29±12.07 | 38.60±15.61 | 46.46±25.26 | 49.67±17.96 |
| Chaoyang | 51 | 123.61±2.39 | 181.40±32.87 | 276.10±26.20 | 28.60±4.56 | 20.10±4.02 | 59.81±8.85 |
| Tongzhou | 8 | 168.18±11.89 | 121.39±41.22 | 124.11±43.10 | 46.12±12.10 | 27.89±5.12 | 37.78±9.02 |
| Daxing | 5 | 247.64±10.65 | 239.64±22.12 | 350.06±25.01 | 32.53±20.29 | 61.76±6.37 | 54.67±13.78 |

Supplementary document 1

Air quality index and air pollution degree in different districts of Beijing within the seven day monitoring period

mean ± standard deviation was reported

Supplementary document 2

Correlation analysis of air quality indicators and concentrations of various air pollutants





Supplementary document 3

Detailed classification of AQI that formulated by China Academy of Environmental Sciences and China National Environmental Monitoring Center

| AQI | Air quality index grade | Air quality index category | Air quality color representation | Health impact |
| --- | --- | --- | --- | --- |
| 0～50 | Grade 1 | Excellent | Green | The air quality is satisfactory and basically free of air pollution |
| 51～100 | Grade 2 | Nice | Yellow | The air quality is acceptable, but some pollutants may have a weak impact on the health of a very small number of extremely sensitive people |
| 101～150 | Grade 3 | Slightly polluted | Orange | The symptoms of susceptible people are slightly aggravated, and healthy people have irritation symptoms |
| 151～200 | Grade 4 | Moderately polluted | Red | Further aggravate the symptoms of susceptible people, which may affect the heart and respiratory system of healthy people |
| 201～300 | Grade 5 | Heavily polluted | Purple | Symptoms of patients with heart disease and lung disease are significantly aggravated, exercise tolerance is reduced, and symptoms are common in healthy people |
| ＞300 | Grade 6 | Seriously polluted | reddish-brown | Healthy people have decreased exercise tolerance, obvious and strong symptoms, and some diseases appear in advance |
